# The Role of Physical Exercise as a Therapeutic Tool to Improve Lipedema: A Consensus Statement from the Italian Society of Motor and Sports Sciences (*Società Italiana di Scienze Motorie e Sportive, SISMeS*) and the Italian Society of Phlebology (*Società Italiana di Flebologia, SIF*)

**DOI:** 10.1007/s13679-024-00579-8

**Published:** 2024-07-03

**Authors:** Giuseppe Annunziata, Antonio Paoli, Vincenzo Manzi, Elisabetta Camajani, Francesco Laterza, Ludovica Verde, Xavier Capó, Elvira Padua, Antonino Bianco, Attilio Carraro, Angela Di Baldassarre, Laura Guidetti, Samuele Maria Marcora, Stefania Orrù, Antonio Tessitore, Roberto Di Mitri, Lucia Auletta, Angela Piantadosi, Mario Bellisi, Edmondo Palmeri, Silvia Savastano, Annamaria Colao, Massimiliano Caprio, Giovanna Muscogiuri, Luigi Barrea

**Affiliations:** 1 Facoltà di Scienze Umane, Della Formazione e dello Sport, Università Telematica Pegaso, Via Porzio, Centro Direzionale, Isola F2, 80143 Naples, Italy; 2 Department of Experimental Medicine, University of Campania “Luigi Vanvitelli”, Naples, Italy; 3 Department of Biomedical Sciences, University of Padua, Padua, Italy; 4 Italian Society of Motor and Sports Sciences, (Società Italiana di Scienze Motorie e Sportive, SISMeS), Verona, Italy; 5 Department of Wellbeing, Nutrition and Sport, Pegaso Telematic University, Centro Direzionale Isola F2, Via Porzio, 80143 Naples, Italy; 6 Department of Human Sciences and Promotion of the Quality of Life, San Raffaele Roma Open University, Rome, Italy; 7 Department of Public Health, University of Naples Federico II, Via Sergio Pansini 5, 80131 Naples, Italy; 8 Translational Research In Aging and Longevity (TRIAL) Group, Health Research Institute of the Balearic Islands (IdISBa), 07120 Palma, Spain; 9 Sport and Exercise Sciences Research Unit, Department of Psychology, Educational Science and Human Movement, University of Palermo, Via Giovanni Pascoli 6, 90144 Palermo, Italy; 10 Faculty of Education, Free University of Bozen-Bolzano, Bozen, Italy; 11 Department of Innovative Technologies in Medicine and Dentistry, “G. d’Annunzio” University of Chieti Pescara, Via dei Vestini 31, 66100 Chieti, Italy; 12 Department Unicusano, University “Niccolò Cusano”, 00166 Rome, Italy; 13 Department of Quality of Life Sciences, University of Bologna, Rimini, Italy; 14 Department of Movement Sciences and Wellness, University Parthenope, 80133 Naples, Italy; 15 Department of Movement, Human and Health Sciences, University of Rome “Foro Italico”, 00135 Rome, Italy; 16 Center for Diagnosis and Treatment of Vascular Diseases, San Rossore Clinic Pisa, Pisa, Italy; 17 Italian Society of Phlebology (Società Italiana Di Flebologia, SIF), Caserta, Italy; 18 “Paolo Giaccone” University Hospital, Palermo, Italy; 19 Serapide Physiotherapy Center – Pozzuoli, (Naples), Italy; 20 Unità di Endocrinologia, Diabetologia e Andrologia, Dipartimento di Medicina Clinica e Chirurgia, Università degli Studi di Napoli Federico II, Via Sergio Pansini 5, 80131 Naples, Italy; 21 Centro Italiano per la cura e il Benessere del Paziente con Obesità (C.I.B.O), Unità di Endocrinologia, Diabetologia e Andrologia, Dipartimento di Medicina Clinica e Chirurgia, Università degli Studi di Napoli Federico II, Via Sergio Pansini 5, 80131 Naples, Italy; 22 Cattedra Unesco “Educazione Alla Salute E Allo Sviluppo Sostenibile”, University Federico II, 80131 Naples, Italy; 23 Laboratory of Cardiovascular Endocrinology, IRCCS San Raffaele, Rome, Italy

**Keywords:** Lipedema, Obesity, BMI, Hormones, Physical activity, Sport

## Abstract

**Purpose of Review:**

This consensus statement from the Italian Society of Motor and Sports Sciences (*Società Italiana di Scienze Motorie e Sportive*, SISMeS) and the Italian Society of Phlebology (*Società Italiana di Flebologia*, SIF) provides the official view on the role of exercise as a non-pharmacological approach in lipedema. In detail, this consensus statement SISMeS - SIF aims to provide a comprehensive overview of lipedema, focusing, in particular, on the role played by physical exercise (PE) in the management of its clinical features.

**Recent Findings:**

Lipedema is a chronic disease characterized by abnormal fat accumulation. It is often misdiagnosed as obesity, despite presenting distinct pathological mechanisms. Indeed, recent evidence has reported differences in adipose tissue histology, metabolomic profiles, and gene polymorphisms associated with this condition, adding new pieces to the complex puzzle of lipedema pathophysiology. Although by definition lipedema is a condition resistant to diet and PE, the latter emerges for its key role in the management of lipedema, contributing to multiple benefits, including improvements in mitochondrial function, lymphatic drainage, and reduction of inflammation.

**Summary:**

Various types of exercise, such as aquatic exercises and strength training, have been shown to alleviate symptoms and improve the quality of life of patients with lipedema. However, standardized guidelines for PE prescription and long-term management of patients with lipedema are lacking, highlighting the need for recommendations and further research in this area in order to optimise therapeutic strategies.

## Introduction

**Fig. 1:**
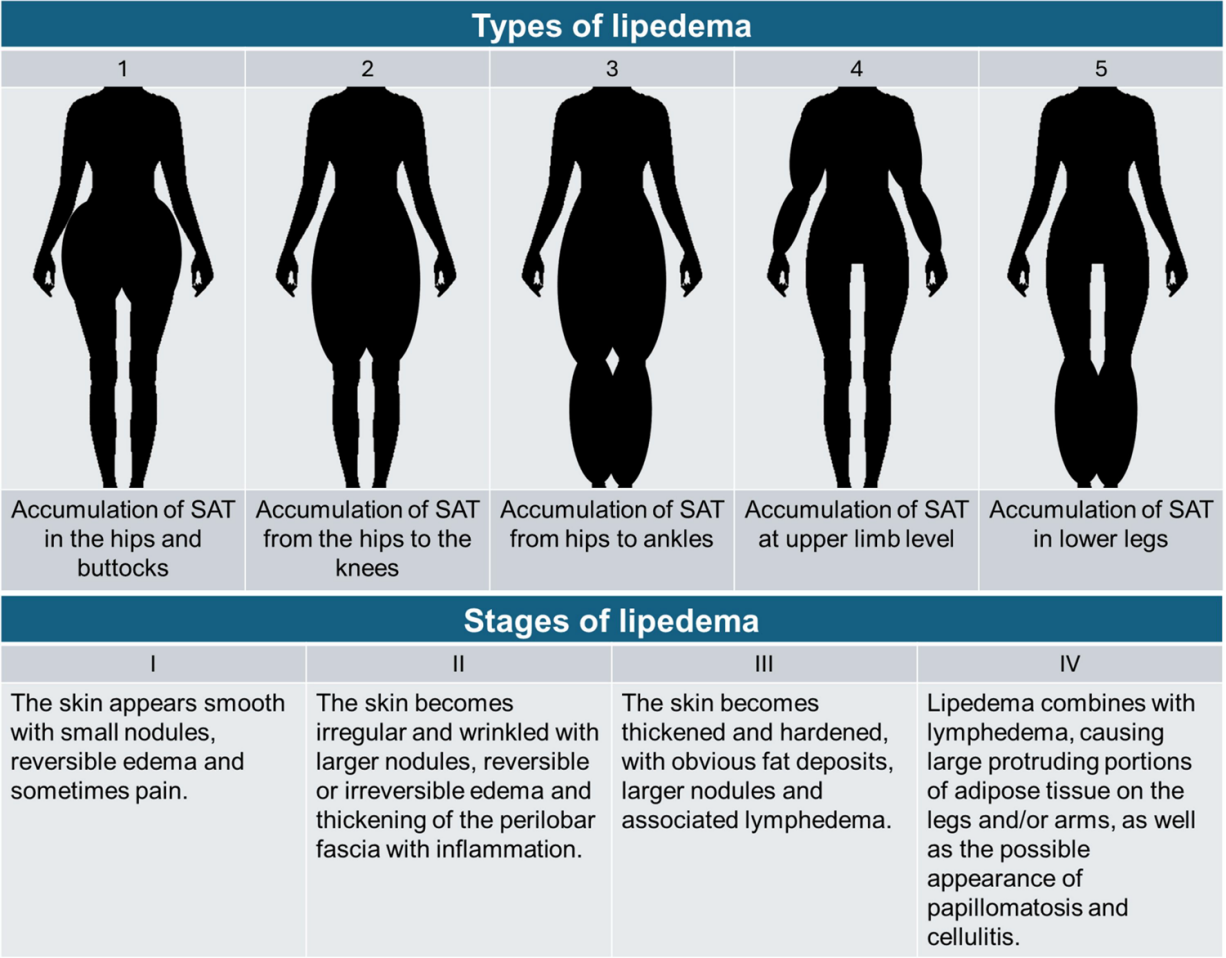
Types and stading of lipedema. Abbreviation: SAT, subcutaneous adipose tissue

Lipedema is a chronic disease of the subcutaneous adipose tissue (SAT) that involves pathological proliferation of adipocytes, especially, but not exclusively, in the lower extremities [1], resulting in a disproportion between the upper and lower parts of the body. In some cases, lipedema may also affect the arms, with a clear dividing line between the hands and feet (the cuff sign) [2]. On objective clinical examination, five different types of lipedema were identified on the basis of the location of SAT accumulations, while a staging of lipedema (stages I-IV) was created on the basis of structural changes in the skin [2] (Fig. 1).

Lipedema is characterized by the symmetrical expansion of the subcutaneous adipose tissue, commonly affecting the lower extremities and, in some cases, the upper extremities [3]. Such SAT expansion, accompanied by increased lipid accumulation within the adipocyte (hypertrophy), is responsible for both the recruitment of immune cells and the rearrangement of the extracellular matrix. These two events result in the beginning of inflammation and in the promotion of pathogenic alterations in vascular and lymphatic functions, which, in turn, result in the accumulation of interstitial fluid and expansion of the interstitial space [2].

It should be remembered that lipedema also needs to be interpreted as a pathology of endocrine interest. In addition to a genetic predisposition, in fact, lipedema initiates due to significant hormonal alterations mainly associated with an abnormal expression of estrogen receptors (ERs) in adipose tissue, in particular characterised by a downregulation of ER-α and an upregulation of ER-β in the affected areas [4, 5]. The altered estrogenic environment fosters aberrant lipid storage patterns, leading to a notable increase in adipocyte size. This phenomenon, coupled with the preferential deposition of fat in specific regions, accentuates the characteristic appearance of lipedema adipose tissue. This suggests the need for a comprehensive view of the pathology and, therefore, the use of multidisciplinary treatments.

To date, the diagnosis of lipedema is still not well standardised and appears challenging, frequently leading to a misdiagnosis of obesity [2]. Several clinical considerations suggest, however, that although lipedema and obesity may coexist, this relationship is not a prerogative, as subjects with normal weight may also be affected by lipedema. In contrast to obesity, indeed, lipedema is mostly characterised by a resistance to losing weight (and fat mass) by resorting to extreme diets or intense exercise [1, 6], which can be frustrating for women with this condition. However, it is speculated that this resistance to diet may have been an evolutionary advantage in women with lipedema since, during periods of famine, they may have accumulated fat (as an energy reserve) to maintain fertility and the ability to breastfeed; this, therefore, would have allowed a continuation of the species [7].

It must be reported that the inability to lose weight through diet can lead to eating disorders and increase the risk of suicide in patients with lipedema [8]. Several complications associated with lipedema, indeed, impair the quality of life of patients suffering from it, including the abnormal accumulation of fat in the lower extremities that alters the gait pattern [9], misaligning the joint axes and causing a valgus deformity of the knee joint [10], and the development of lymphoedema observed in some cases of lipedema, leading to further mobility limitations, further worsening patients' quality of life [11]. In previous studies, it was observed that leg discomfort, consisting of widespread pain, tenderness, and painful anguish, was a prominent symptom in about half of the subjects with lipedema [6]. Other notable signs and symptoms that are more frequent in patients with stage 2 and 3 lipedema than in stage 1 are obstructive sleep apnoea, blood clots, nausea, constipation, increased body temperature, flu-like symptoms, and burning skin pain [12]. This condition, therefore, leads people to suffer from a low quality of life and develop psychological signs of depression [13].

From a nutritional point of view, it has been previously reviewed that studies on different types of diets can shed light on this aspect of the disease [7]. In general, a ketogenic dietary approach (i.e., low-carbohydrate high-fat diets) appears to be effective in promoting weight (and fat mass) loss in women with lipedema, acting through some metabolic changes (i.e., reduction of basal insulin levels and HoMA-IR index), and reducing inflammation and oxidative stress [2, 14, 15].

This implies, therefore, the need to find effective strategies for the management of women with lipedema in order to improve both nutritional status and quality of life. In this sense, many conservative treatments to alleviate symptoms and increase quality of life include physical exercise (PE) [16], which, however, is not always feasible due to the debilitating clinical condition. This may lead to muscle weakness, as reported in subjects with lipedema [16]. However, it is not fully clear whether the observed weakness is part of the pathology or is caused by decreased physical activity (PA). Furthermore, decreased PA has been correlated with increased symptom complaints, muscle weakness, fatigue, and weight gain [16].

This consensus statement from the Italian Society of Motor and Sports Sciences (*Società Italiana di Scienze Motorie e Sportive*, SISMeS) and the Italian Society of Phlebology (*Società Italiana di Flebologia*, SIF) provides the official view on the role of exercise as a non-pharmacological approach in lipedema. In detail, this consensus statement SISMeS—SIF aims to summarise the available literature in order to provide an overview of lipedema, its complications, and phenotypes, focusing on the role that PA plays in improving the clinical features of this disabling condition, as well as to identify the optimal prescription of PA for this class of patients.

## Lipedema: Not Just a BMI Matter

Lipedema is often confused with obesity and has not, until now, been considered a distinctive phenotype of obesity. Genetic analyses may, therefore, be crucial to distinguish lipedema from genetic obesity, primary lymphoedema and lipodystrophies [17–19]. It is important to note, however, that lipedema can coexist with obesity, but it should not be confused with this metabolic disease [20–22]. In fact, this condition differs from severe obesity in several aspects: (i) it mainly affects women [23, 24], (ii) although many patients are with overweight, many women with lipedema have a normal weight and present disproportionately enlarged lower extremities, (iii) and patients with overweight and lipedema do not see any decrease in the size of the lower extremities with diet or weight loss [24]. This evidence suggests, therefore, that other parameters besides body weight and body mass index (BMI) need to be monitored for early assessment and management of patients with lipedema, since this condition can also occur in women with normal body weight. As well, in addition to BMI, other parameters should be taken into account for the differential diagnosis.

### Beyond the BMI

In the case of lipedema, BMI may not accurately reflect the adiposity index. This is because BMI is based only on the measurement of weight and height, without distinguishing between different types of adipose tissue or considering the distribution of body fat. Therefore, in the context of lipedema, it is important to consider other measurements and clinical evaluations to accurately assess the body composition and general health of the patient, as discussed above. In this sense, Brenner and colleagues conducted a retrospective study on more than 600 women with a diagnosis of lipedema, proposing the use of the Waist-to-Height Ratio (WHtR) as an alternative [25]. The authors pointed out that WHtR is independent of total weight, thereby not influenced by variations in leg or arm weight, and offers a more accurate reflection of nutritional condition, resulting in a more suitable measure for assessing metabolic risks in research studies. The main aim of Brenner’s study was the identification of WHtR values in patients with lipedema who were also classified as with overweight or obesity. In addition, they compared the WHtR values with those of the general population [25], using a German study of over 5000 women as a reference [26]. The study highlighted a limitation in using BMI alone to evaluate patients with lipedema, suggesting that relying solely on BMI not only overestimates metabolic risks but can also result in misdiagnosing obesity due to the disproportionate distribution of SAT characteristic of lipedema. The authors, thus, recommended using WHtR as an alternative measure to evaluate or rule out obesity in lipedema patients. They further suggested that if both BMI and WHtR are utilized, a normal WHtR alongside an increased BMI indirectly indicates a disproportionate increase in SAT. However, they clarify that neither BMI nor WHtR are diagnostic tools for lipedema [25].

**Fig. 2:**
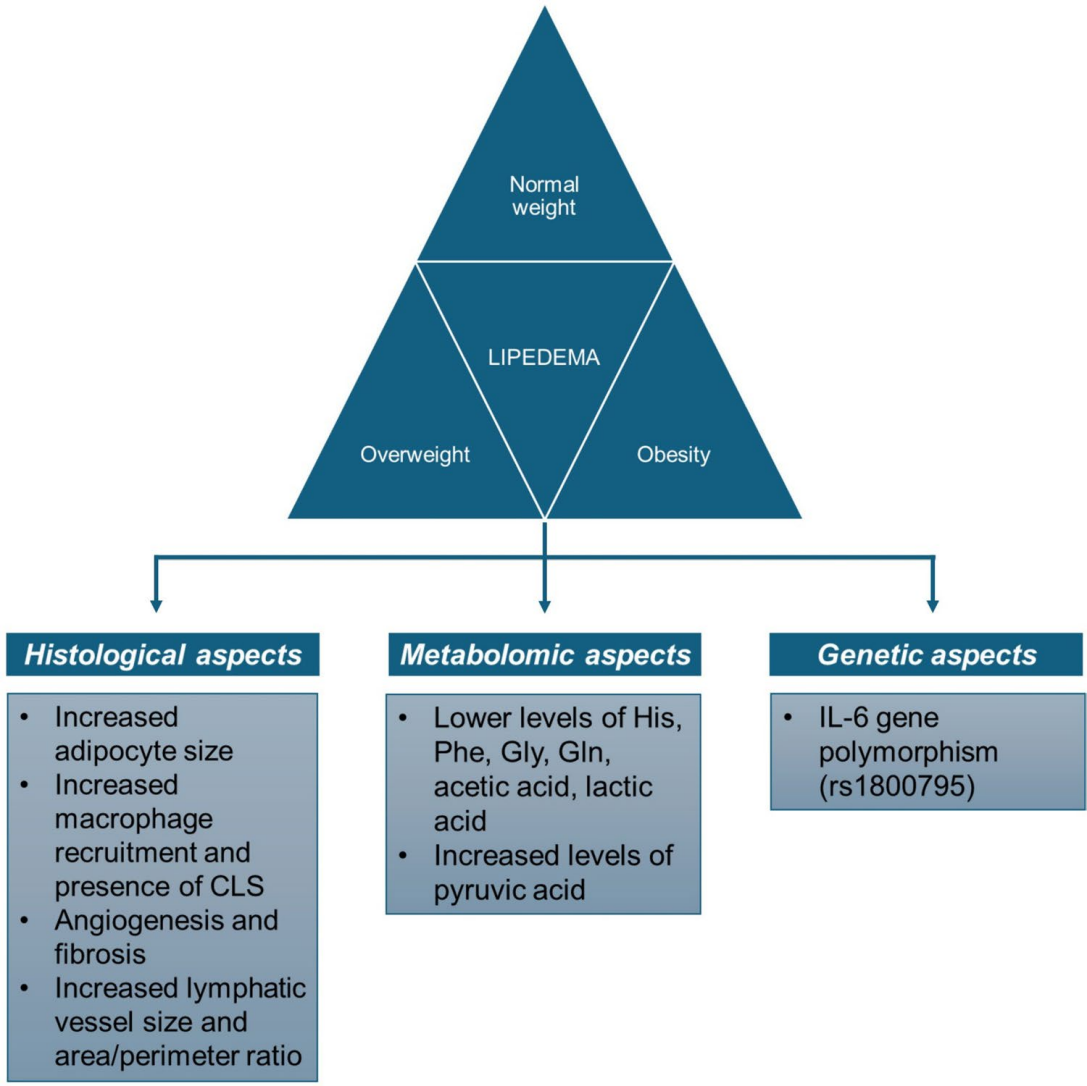
Lipedema, beyond BMI. The main features commonly present in patients with lipedema, independently of body weight and BMI. Abbreviation: CLS, crown-like structures; His, Histidine; Phe, Phenylalanine; Gly, Glycine; Gln, glutamine; IL, interleukin

Having clarified that the use of BMI may be inadequate, or at least inaccurate, for subjects with lipedema, interesting evidence adds a new piece to the complex puzzle of the pathophysiology of lipedema, suggesting the existence of common clinical features in this class of patients that are independent of body weight and BMI, including SAT histology [27], metabolomic profile [28], and gene polymorphisms [29] (Fig. 2).

### Histological Differences in SAT

As already mentioned, the main characteristic of lipedema is the symmetrical expansion of the SAT, which is commonly found in the lower limbs and, in some cases, in the upper limbs [3]. This expansion of the SAT, together with a hypertrophic condition, results in both the recruitment of immune cells and the rearrangement of the extracellular matrix, which, taken together, are responsible for the establishment of an inflammatory state and the promotion of pathogenic changes in the vascular and lymphatic functions; these, in turn, promote the accumulation of interstitial fluid and the expansion of the interstitial space [2].

Al-Ghadban and colleagues conducted a study to compare lipedema SAT to non-lipedema SAT in groups of 49 women (30 with lipedema and 19 without), categorised into obesity (BMI 30.0 to 40.0 kg/m^2^, Ob) and non-obesity (BMI 20.0 to 30.0 kg/m^2^, N-Ob) [27]. Histologically, authors observed heterogeneity in adipocyte size in both Ob and N-Ob lipedema groups, but significant increased cell size in the N-Ob lipedema (NOL) group compared to the N-Ob control (NOc) group. This suggests that adipocyte size heterogeneity is not a reliable marker of lipedema fat, but adipocyte hypertrophy in lipedema occurs independently of obesity. Furthermore, an increase in macrophages in both skin and fat was observed in the NOL group, which resembles the results in non-lipedema obesity. The presence of crown-like structures (CLS) in lipedema fat, typically indicative of metabolically poor tissue, was noted regardless of obesity status. Angiogenesis was observed in the skin of NOL women, with increased blood vessel numbers in the papillary dermal layer, possibly indicating underlying angiogenesis in lipedema. In addition, NOL women exhibited significant capillary dilation in fat compared to controls, along with evidence of angiogenesis and fibrosis. The authors also noted variations in lymphatic vessel morphology with Ob-lipedema (OL), women showing an increase in vessel size and area/perimeter ratio. Overall, the study provides insights into the histological characteristics of lipedema fat, highlighting similarities and suggesting potential markers for disease progression [27].

### Metabolomic

The application of metabolomic in lipedema research in order to identify complex metabolic alterations and potential biomarkers for early diagnosis, prognosis, and treatment strategies was recently evaluated in an interesting study conducted on groups of 25 women with lipedema, 25 women with obesity, and 25 normal weight women [28]. The main results revealed that the metabolic and lipidomic profiles of patients with lipedema differed significantly from those of controls without lipedema. Among the 39 metabolites examined, nine were significantly altered in lipedema. In particular, patients with lipedema showed lower levels of histidine and phenylalanine, whereas pyruvic acid was elevated in comparison to controls. Furthermore, histidine, phenylalanine, and pyruvic acid concentrations showed promising diagnostic accuracy in distinguishing patients with lipedema from those with obesity but without lipedema, with pyruvic acid showing the most promise. Subgroup analysis within BMI ranges indicated that differences in pyruvic acid, phenylalanine, and histidine levels are likely to be associated with lipedema rather than changes in BMI [28]**.** These findings provide important insights into the metabolic alterations associated with lipedema. In particular, according to the authors, the lower levels of histidine and phenylalanine found in women with lipedema could indicate alterations in protein metabolism, amino acid utilization, and related metabolic pathways. Since histidine and phenylalanine are precursors of essential molecules [28], such as hormones and neurotransmitters [30, 31], their disruption could affect crucial processes such as protein synthesis, energy production, and neurotransmitter regulation. These changes suggest potential alterations in amino acid metabolism in lipedema patients [28]. Pyruvate is a key molecule in adipocyte metabolism [32], contributing to several crucial processes. These include energy production, triglyceride synthesis, fatty acid production, and glucose regulation [33, 34]. In Kempa's study, the authors observed a weak correlation between pyruvate levels and BMI, suggesting that lipedema, per se, might increase pyruvate levels independently of BMI [28]. Pyruvate metabolism is tightly regulated to meet the energy and storage demands of adipocytes in response to nutritional and physiological stimuli [35]. An alteration in pyruvate metabolism in adipocytes can lead to the accumulation of triglycerides [35]. In fact, the availability of acetyl-CoA derived from pyruvate is essential for the de novo synthesis of fatty acids [36], which, despite its minimal contribution, may influence some of the metabolic alterations observed in lipedema. In addition, according to Kempa and colleagues, the presence of high pyruvate levels in lipedema could indicate an alteration in the citric acid cycle. If pyruvate is not utilized effectively in the Krebs cycle, it accumulates, causing its high levels. The researchers also observed low levels of acetic acid, glycine, glutamine, and lactic acid in the lipedema group, suggesting dysfunction in various metabolic pathways [28]. The decrease in acetic acid could indicate an alteration in lipid metabolism, particularly in the oxidation or synthesis of fatty acids [37]. In addition, acetic acid plays a role in body weight regulation and adipose tissue function [38]. As for glycine, its serum levels correlate positively with SAT and negatively with visceral adipose tissue (VAT) [39], suggesting a potential involvement of this amino acid in the distribution of adipose tissue, understanding of the underlying mechanisms of which would provide insight into the different phenotypes of lipedema (e.g. hyperglycolytic phenotype showing serine and glycine overproduction), as well as guide potential lines of treatment, such as glycine supplementation [28]. Glutamine, on the other hand, is implicated in energy production [40], nitrogen metabolism [41], and neurotransmitter synthesis [42], as well as exerting an action in reducing adipose tissue and inflammation [43], so a decrease in its levels may reflect changes in these processes. A reduction in lactate levels could signal a change in metabolism, both glycolytic and oxidative [44]. Lactic acid has several functions and has been observed to promote the transformation of adipocytes into beige adipocytes [45], which, by expressing high levels of uncoupling protein 1, produce heat [46]. Finally, differences in LDL-transported lipids were also observed in patients with lipedema compared to controls, suggesting an association with lipedema pathology rather than adiposity [28]. Metabolomics, thus, presents itself as a valuable tool for understanding metabolic alterations in lipedema and investigating the underlying mechanisms [28].

### IL-6 Gene Polymorphism

The clinical study conducted by Di Renzo and colleagues identified new indices and predictive parameters based on body composition and IL-6 gene polymorphism (rs1800795) that could distinguish individuals with lipedema from those with normal weight-obesity (NWO) and obesity [29]. This study examined the complex genetic interactions related to body fat accumulation in patients with lipedema and found significant differences in fat distribution in women carrying or not carrying the IL-6 gene polymorphism (rs1800795). In particular, being a carrier of the mutation increases the risk of developing lipedema by almost 6-fold [29]. IL-6 plays an important role in regulating body fat, as it is released from adipocytes and its levels are elevated in obesity, indicating the presence of an inflammatory state [47–49]. The IL-6 gene polymorphism (rs1800795), which affects IL-6 transcription, has been identified as a cause of the onset of overweight [47]. Previous studies have also shown that IL-6 concentration correlates positively with fat mass percentage in the GG genotype and negatively in the CC genotype [47–49]. The authors suggest, therefore, that the leg index, abdominal index, trunk index, and total index, combined with genetic analysis of the IL-6 gene polymorphism (rs1800795), can be used as promising clinical tools to diagnose the phenotype of lipedema and predict the evolution of the disease [29].

## An Endocrine Outlook for Lipedema

Emerging evidence suggests potential links between lipedema and hormonal influences [50]. This section aims to elucidate the complex interplay between estrogens, adipose tissue, and the effects of PE, offering novel insights for tailored therapeutic interventions.

Estrogens exert multiple effects on adipose tissue, contributing to body fat distribution and adipose depot remodeling, mostly mediated by ERα; they positively influence mitochondrial function and curb inflammation [51]. Alterations in estrogen activity or the lack of estrogen receptors (ERs) result in the accumulation of subcutaneous adipose tissue (SAT), a phenomenon observed in patients with lipedema [52]. Furthermore, according to Al-Ghadban, ERα knockout mice have shown that a reduction in estrogen resulted in increased adipose tissue inflammation with the upregulation of pro-inflammatory markers, such as interleukins IL-1β, IL-6, and tumor necrosis factor-alpha [53].

Moreover, estrogens act as central mediators for food intake and energy consumption in the hypothalamus [50]. The site-specific localization of adipose tissue, especially in the lower limbs of women with lipedema, appears strictly linked to estrogen levels [2].

Women with lipedema often exhibit notable alterations in ER expression, with a predominant focus on ERα [54]. Dysregulation in ERα, characterized by aberrant expression levels or impaired signalling pathways, disrupts the finely tuned balance maintained by estrogen in adipose tissue. These receptor-specific alterations play a pivotal role in deciphering the pathophysiology of the condition [54].

According to Katzer and colleagues, ERα and ERß may play a role in the dysregulated adipose tissue characterized by lipedema, and the proposed estrogen-mediated dysregulation, associated with the characteristic accumulation of excessive SAT in the lower body characteristic of lipedema, could operate through two distinct mechanisms [54]. In particular, some authors hypothesised that the adipocytes of subjects with lipedema may display a higher ERα/ERβ expression ratio than in subjects without lipedema [54]. This could lead to lower ERβ-induced suppression of ERα-mediated gene expression, reduced inhibition of ERβ, resulting in increased activation of PPARγ by ERα, elevated entry of free fatty acids into adipocytes for triacylglycerol production through increased lipoprotein lipase activity, decreased lipolysis due to increased lipoprotein lipase activity, a decrease in lipolysis due to ERα-induced upregulation of anti-lipolytic α-adrenoreceptor, ERα-induced increase in glucose uptake through enhanced insulin-stimulated GLUT4 translocation, an increase in angiogenesis through ERα-induced upregulation of VEGF, and finally a decrease in mitochondriogenesis and mitochondrial function [54].

The GH/IGF1 pathway displays profound effects on adipocyte metabolism, given that GH is a powerful stimulator of lipolysis; such effects are not mediated by IGF-1, whereas IGF-1 is a pivotal regulator of the terminal phases of adipose cell differentiation. So far, no studies have investigated the GH/IGF-1 axis in patients with lipedema; interestingly, an in vitro study performed on adipose stem cells obtained from lipoaspirate demonstrated a higher expression of IGF-1 during the proliferative activity in stem cell cultures from patients with lipedema in comparison with control stem cells [55].

It is well known that the prevalence of hypothyroidism is higher in women than in men [56] and in patients with obesity than in normal weight subjects [57]. For these reasons, thyroid status could be easily altered in lipedema. In fact, in women affected by lipedema, a higher prevalence of hypothyroidism than in the normal population has been reported by more studies [55, 58] with a progressively higher increase with the severity of the clinical stage. Similarly, the prevalence of autoimmune thyroiditis in female patients with lipedema was higher than in the normal population [55].

## Lipedema and Physical Exercise

### The Multiple Beneficial Effects of Physical Exercise on Lipedema

PE emerges as a cornerstone in the multifaceted management of lipedema [59]. PE, particularly endurance training, indeed, stands out as a potent modulator of mitochondrial function within SAT [60]; the enhancement of mitochondrial activity through PE may be considered an effective therapeutic tool for individuals with lipedema, contributing to improved lipid metabolism and potentially countering aberrant lipid storage patterns. A compelling aspect of PE in the context of lipedema lies in its potential to modulate inflammation and lipolysis [60]. An expanding body of evidence has consistently demonstrated a general and white adipose tissue (WAT)-related, anti-inflammatory impact of chronic exercise training [61]. Before recognizing such an exercise-induced effect, specifically in WAT, it had already been established that exercise training leads to a decrease in circulating inflammatory markers. This effect has been observed not only in people with obesity but also in subjects with increased inflammation.

It is worthy to mention that acute exercise exerts, instead, a temporary increase of inflammatory cytokines necessary to stimulate the exercise adaptation mechanisms related to performance and physical fitness improvement [62]. Given the robust connection between inflammation in WAT and systemic inflammation, it is reasonable to hypothesize that PE may directly influence the inflammatory status of WAT [61]. Interestingly, PE training promotes the browning of WAT *via* multiple mechanisms, one of the most attractive of which is the increasing levels of the myokine irisin [63, 64]. Specifically, PE stimulates PGC-1α, which, in turn, upregulates the expression of fibronectin type III in skeletal muscle. The cleavage of fibronectin III domain 5 releases irisin, which, upon entering the circulation, reaches the adipose tissue, where it promotes WAT browning through an increase in Ucp1 mRNA and the number of Ucp1-positive cells [63, 64]. Regular PA exerts a suppressive influence on the pro-inflammatory milieu observed in lipedema SAT. This anti-inflammatory effect, attributed to increased catecholamine secretion and alternative macrophage activation, represents a critical pathway through which PE enhances adipose tissue health [10]. There is, in fact, evidence indicating that PE induces the phenotypic switch from M1 to M2 macrophages in adipose tissue, particularly in subjects with obesity. More specifically, it has been shown that PE inhibits the infiltration of M1 macrophages into the adipose tissue and increases the expression of CD163, a specific marker of M2 macrophages. This macrophage phenotypic switching contributes to inhibiting the chronic inflammatory state in adipose tissue [65]. Also, PE orchestrates additional beneficial changes within lipedema SAT by promoting angiogenesis and augmenting antioxidant defences. Improved vascularization in muscle induced by PE enhances tissue perfusion and oxygenation, potentially mitigating hypoxic conditions observed in advanced lipedema stages. Furthermore, the boostering of antioxidant defences aligns with the broader goal of enhancing tissue resilience and mitigating oxidative stress [10]. Pioneering studies by Stallknecht in 1991 demonstrated PE-induced improvements in mitochondrial function within adipocytes, suggesting PE's broader impact on adipose tissue health [66].

PE positively influences adipose tissue health through mechanisms such as exosome and myokine release [67–70], catecholamine release, AMPK activation, and angiogenesis. Exploring how these mechanisms interact with hormonal modifications in the context of lipedema is important for developing novel therapeutic strategies. According to the standard of care for lipedema in the United States, people with lipedema may benefit from postural and core exercises, muscle strengthening exercises, gait training, neuromuscular re-education, and deep abdominal breathing to increase lymphatic flow and stimulate the parasympathetic system [58]. Beyond weight management, PE positively influences adipocyte health. Moreover, a recent review by Esmer and colleagues confirms that complex decongestive physiotherapy, gait training, hydrotherapy, aerobic exercise, and resistance exercise training are all effective in the management of lipedema [10].

Many authors suggest the importance of regular PE in the management of lipedema. In particular, self-management techniques emphasise the importance of low-impact PE for lipedema sufferers, taking into account the patient's preferred activities [71]. Furthermore, the importance of increasing muscle strength in the conservative treatment of lipedema is emphasised [16]. PE, in fact, not only counteracts the main symptoms, such as fatigue and muscle weakness, but is crucial to encourage patients to include PA as part of their daily routine in order to maintain a healthy lifestyle [16]. As another important positive effect of PE, exercises involving the leg and calf muscles have been shown to increase lymphatic drainage and venous flow, reducing or preventing oedema [72]. This insight adds another valuable piece to the mosaic of the multiple benefits of PE in the management of lipedema [10]. Poor adherence to PE and PA could be solved by selecting graded exercises [73] and manipulating intrinsic and extrinsic motivation, where intrinsic refers to the individual's enjoyment of performing the activity, while extrinsic motivation is related to tangible benefits such as material or social rewards, or to avoid punishment [74]. By studying these psychological aspects, it is possible to structure a tailor-made conservation programme that could also be extended to a healthy lifestyle in general.

Psychological problems associated with lipedema patients, such as depression and anxiety [10], could be managed with PE [75]. According to several authors, after aerobic exercise, participants show increased pressure-pain tolerance [76–78]. Consequently, these forms of PE could play a key role in addressing the pain characteristic of patients with lipedema [59].

The PE effect is also crucial for other signs and symptoms found most frequently in patients with stage 2 and 3 lipedema, such as obstructive sleep apnea [12]. Indeed, it has been shown that PE can be beneficial for the management of  obstructive sleep apnea, beyond simple weight loss [79].

### The Optimal Physical Exercise Prescriptions

As described above, the main goal of lipedema treatments is to manage pain and reduce functional limitations due to excessive limb volume. However, lipedema patients do not respond to caloric restriction and intense PA [6, 80], so conservative treatments, including PE, are proposed to alleviate the symptoms and improve the quality of life.

In the literature, some studies have included PE in conservative treatments to alleviate the symptoms associated with lipedema; Table 1. Atan & Bahar-Özdemir and colleagues [81], compared the effectiveness of three different conservative treatments. Patients were randomised into three groups: Group 1 (Complete Decongestive Therapy (CDT) plus PE), Group 2 (intermittent pneumatic compression therapy (IPCT) plus PE), and Group 3 (control PE-only). The training protocol was structured as follows: 5 days a week for 6 weeks, with each workout consisting of a warm-up, aerobic exercises, strengthening exercises, and stretching. The aerobic section was individualised, prescribing the same heart rate achieved at the end of the 6-minute walk test, which was approximately 80% of the theoretical maximum heart rate. Strengthening exercises, on the other hand, consisted of resistance exercises for the major muscle groups. The main findings were that limb volume measurements decreased significantly in the CDT group compared to the other two groups and that IPCT applied in addition to PE was not superior to the PE-only group in patients with severe lipedema [81]. In another study [82], the therapeutic potential of physical therapy was evaluated by prescribing a 60-minute therapeutic session. The session consisted of manual therapy (including manual lymphatic drainage) and a customised PE guide that provided guidance on posture, joint protection, movement and PE, compression requirements, diaphragmatic breathing, and healthy eating. The PE programme was customised and aimed at lower-body strengthening, flexibility, and conditioning, focusing on long-term adherence. Over the 6-week protocol period, the results reported a decrease in pain perception, as assessed by the visual analogue pain scale, an improvement in quality of life, and a tendency to sodium reduction, as assessed by MRI [82]. However, it should be emphasised that, as designed, the study by Donahue and colleagues [82] does not allow unequivocal discrimination as to which was individually the effect of PE and which was that of manual therapy.

Research evaluating the effects of aquatic exercise is more frequent. Amato and Benitti [83] studied the use of aquatic exercise combined with diet, lymphatic drainage, and antioxidant herbal drugs in five cases of lipedema assessed with the Lipedema Symptom Assessment Questionnaire, one for each stage of the disease. The results showed a positive effect of these treatments [83]. The lack of specificity of the applied aquatic PEs, however, calls for further investigation. Again, no conclusions can be drawn about the effect of PE on lipedema.

Overall, experimental studies that have applied PEs to lipedema are limited; however, many guidelines suggest their possible application based on the rationale of the disease. The guidelines by Reich-Schupke et al. [59] and Kruppa et al. [4] recommend PA, particularly emphasising the effectiveness of water-based exercises such as swimming, aqua jogging, and water aerobics. Floating in water relieves joint pressure, promotes lymphatic drainage, and contributes to calorie burning, making it a favourable option for the management of lipedema [59]. Aquatic activities might be suitable for patients who complain of worsening lower-limb oedema at the end of the day, often related to orthostasis and heat [84]. In this sense, Gianesini et al. [85] designed a specific aquatic protocol that demonstrated a positive impact on chronic leg swelling. Table 1 shows a summary of studies that have analyzed the effect of exercise on lipedema patients.

**Table 1:** Summary of the studies that have analysed the effect of exercise, often combined with other conservative therapies, on patients affected by lipedema

**Ref.**	**Exercise proposed**	**Variables analysed**
[6]	- Cyclical walking - Running movements - Swimming /aqua jogging/ aqua gymnastic	Guideline
[3]	- Swimming - Aqua jogging-aerobics-cycling	Guideline
[76]	- Regular aquatic physical exercise	• QuASiL questionnaire • Volumes and proportions
[75]	- Tailored exercise programs addressed lower body strengthening, flexibility, and conditioning, focusing on long-term adherence.	• Tesla sodium and water MRI • Biophysical measurements • Questionnaires measuring function • Questionnaires QoL (patient-specific functional scale, PSFS, and RAND-36) • VAS
[15]	- increasing muscle strength in conservative treatment for lipedema	• Muscle strength of the quadriceps was assessed with dynamometer • 6-min walk test
[74]	- Warm-up - Aerobic exercise at 80% of the maximal theoretical heart rate - Strengthening exercises	• Limb volume measurements • Anthropometric measurements • 6-min walk test • VAS • fatigue severity scale • Beck Depression Inventory • Short Form Health Survey-36
[79]	- Water sports	• VAS • DLQI • Health-related quality of life according to Short Form 36 (SF-36) • World Health Organization Quality of Life-abbreviated version of the WHOQOL 10 (WHOQOL-BREF) • Patient Health Questionnaire-9 (PHQ-9) • Leg circumference • Lower • Extremity Functional Scale, LEFS • Body fat percentage • Hematoma tendency • Occurrence of leg oedema
[4]	- Weight loss exercise schedule	Guideline

Guidelines suggested the activity described considering the typical symptoms and characteristics of the disease

*QuASiL* Questionário de Avaliação Sintomática do Lipedema*, MRI* magnetic resonance imaging*, QoL* Quality of Life*, PSFS* Patient-Specific Functional Scale*, VAS* Visual analogue scale, *DLQI* Dermatology Life Quality Index*, LEFS* Lower Extremity Functional Scale

## Conclusion

This is a consensus statement from the Italian Society of Motor and Sports Sciences (*Società Italiana di Scienze Motorie e Sportive,* SISMeS) and the Italian Society of Phlebology (*Società Italiana di Flebologia,* SIF) which provides the official view on the role of exercise as a non-pharmacological approach in lipedema.

**Fig. 3:**
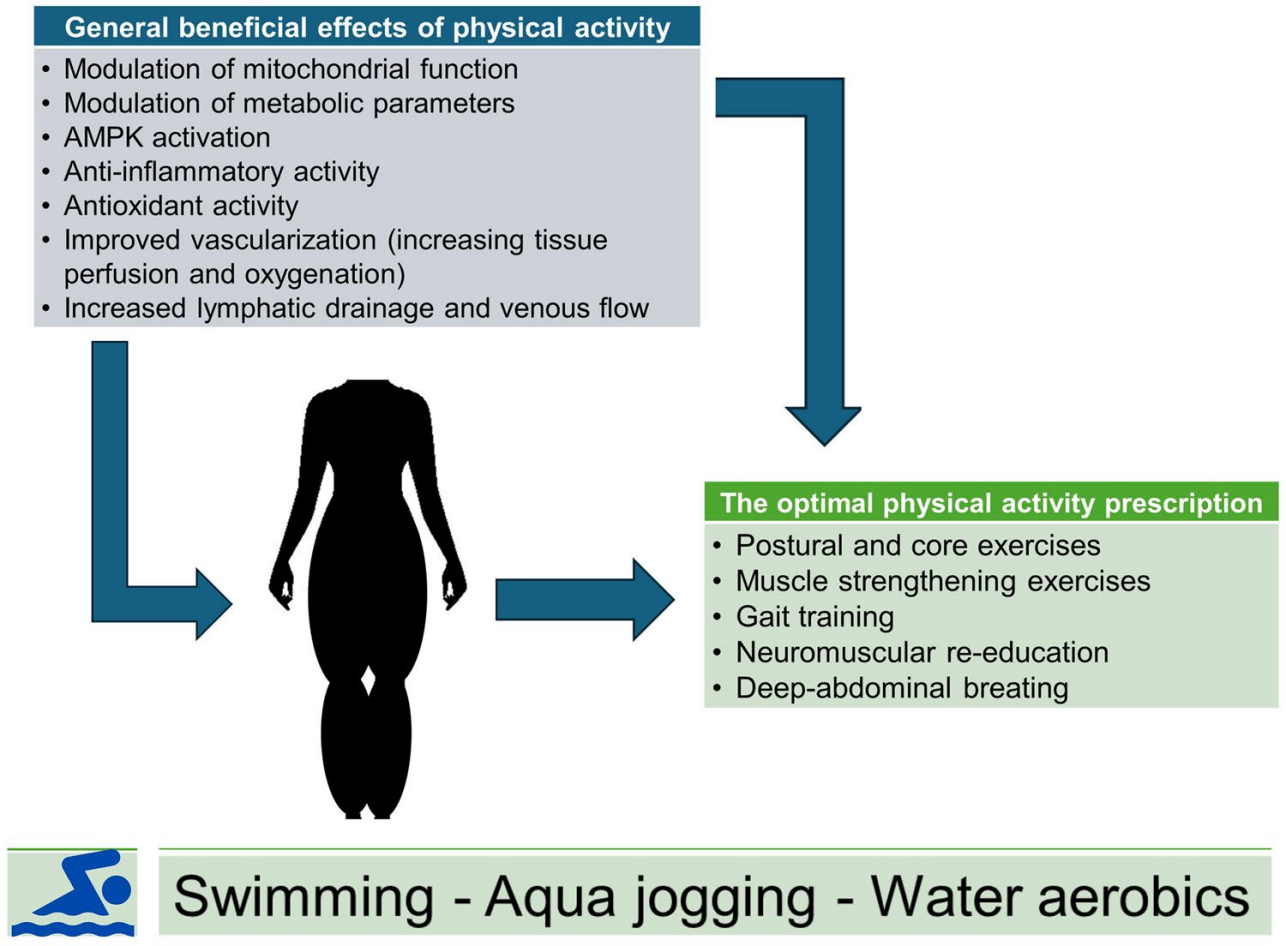
PE as a therapeutic tool to improve lipedema

Lipedema is a particularly complex and multifactorial condition that requires, at its base, a careful diagnosis and, above all, a multidisciplinary approach for its management that takes into account the etiopathogenetic, endocrine-metabolic, and nutritional aspects. Treatments must, inevitably, consider the pathology from a holistic prespective, and be appropriately tailored to the patient's needs. In this perspective, PE plays a major role (Fig. 3). The body of available literature emphasises the potential benefits of PE in the holistic management of lipedema. Structured exercise training, particularly water exercise, emerges as a promising intervention, offering not only physiological benefits such as weight control, functional activity, joint relief, and lymphatic drainage but also psychological benefits, with a positive impact on self-esteem, mood, and quality of life. Tailor-made PE programmes, including muscle strengthening, flexibility training, and whole-body conditioning, are essential for conservative treatment plans. However, the literature reveals the need for further studies on a larger number of subjects to establish standardised PE prescriptions tailored to the different stages of lipedema, improving our understanding of optimal treatment approaches and ultimately improving the quality of life of people struggling with this difficult condition.

## Data Availability

No datasets were generated or analysed during the current study.
